# Identifying Bacterial Airways Infection in Stable Severe Asthma Using Oxford Nanopore Sequencing Technologies

**DOI:** 10.1128/spectrum.02279-21

**Published:** 2022-03-24

**Authors:** Maisha F. Jabeen, Nicholas D. Sanderson, Dona Foster, Derrick W. Crook, Jennifer L. Cane, Catherine Borg, Clare Connolly, Samantha Thulborn, Ian D. Pavord, Paul Klenerman, Teresa L. Street, Timothy S. C. Hinks

**Affiliations:** a Respiratory Medicine Unit, Experimental Medicine Division, Nuffield Department of Medicine, University of Oxford, John Radcliffe Hospital, Oxford, United Kingdom; b Nuffield Department of Clinical Medicine, University of Oxford, John Radcliffe Hospital, Oxford, United Kingdom; c National Institute for Health Research Oxford Biomedical Research Centre, John Radcliffe Hospital, Oxford, United Kingdom; d Peter Medawar Building for Pathogen Research and Translational Gastroenterology Unit, Nuffield Department of Clinical Medicine, University of Oxford, Oxford, United Kingdom; Nanchang University

**Keywords:** asthma, bacteria, *Haemophilus influenzae*, Illumina, microbiome, Oxford Nanopore, sputum

## Abstract

Previous metagenomic studies in asthma have been limited by inadequate sequencing depth for species-level bacterial identification and by heterogeneity in clinical phenotyping. We hypothesize that chronic bacterial airways infection is a key “treatable trait” whose prevalence, clinical phenotype and reliable biomarkers need definition. In this study, we have applied a method for Oxford Nanopore sequencing for the unbiased metagenomic characterization of severe asthma. We optimized methods to compare performance of Illumina MiSeq, Nanopore sequencing, and RT-qPCR on total sputum DNA extracts against culture/MALDI-TOF for analysis of induced sputum samples from highly phenotyped severe asthma during clinical stability. In participants with severe asthma (*n* = 23) H. influenzae was commonly cultured (*n* = 8) and identified as the dominant bacterial species by metagenomic sequencing using an optimized method for Illumina MiSeq and Oxford Nanopore. Alongside superior operational characteristics, Oxford Nanopore achieved near complete genome coverage of H. influenzae and demonstrated a high level of agreement with Illumina MiSeq data. Clinically significant infection was confirmed with validated H. influenzae plasmid-based quantitative PCR assay. H. influenzae positive patients were found to have sputum neutrophilia and lower FeNO. In conclusion, using an optimized method of direct sequencing of induced sputum samples, H. influenzae was identified as a clinically relevant pathogen in severe asthma and was identified reliably using metagenomic sequencing. Application of these protocols in ongoing analysis of large patient cohorts will allow full characterization of this clinical phenotype.

**IMPORTANCE** The human airways were once thought sterile in health. Now metagenomic techniques suggest bacteria may be present, but their role in asthma is not understood. Traditional culture lacks sensitivity and current sequencing techniques are limited by operational problems and limited ability to identify pathogens at species level. We optimized a new sequencing technique—Oxford Nanopore technologies (ONT)—for use on human sputum samples and compared it with existing methods. We found ONT was effective for rapidly analyzing samples and could identify bacteria at the species level. We used this to show Haemophilus influenzae was a dominant bacterium in the airways in people with severe asthma. The presence of Haemophilus was associated with a “neutrophilic” form of asthma - a subgroup for which we currently lack specific treatments. Therefore, this technique could be used to target chronic antibiotic therapy and in research to characterize the full breadth of bacteria in the airways.

## INTRODUCTION

The field of airway metagenomics has expanded with wider application of bacterial 16S rRNA sequencing, utilizing Roche/454 pyrosequencing or Illumina MiSeq platforms to profile the structure of the microbiota (1–4). Rapid developments in characterization and understanding of the lung microbiome in chronic airways disease offer growing opportunities to incorporate these findings within the “treatable trait” paradigm for disease management now considered the gold standard of care in asthma (5), which globally remains the commonest chronic lung disease. This is particularly critical for the 5–10% of individuals affected by severe, treatment-refractory asthma displaying the greatest clinical and immunopathological heterogeneity, with high exacerbation risk and mortality (6). In several reports Haemophilus influenzae has been identified as the commonest potentially pathogenic organism in the airway of severe asthmatics in stable disease, associated with sputum neutrophilia and altered microbial diversity, namely, reduced Streptococcus, Gemella, and Porphyromonas taxa (2, 7, 8). Sputum neutrophilia correlates with bacterial burden and type-1 cytokines (4, 9), with positive sputum culture present in up to 43% of patients (10). In severe asthma overall, potentially pathogenic organisms have been isolated by culture in 20–52% (11, 12). Metagenomic studies are however limited by either small sample-size (1, 7) and inadequate clinical phenotyping of a heterogenous population, or insufficient sequencing depth and inability to reliably assign taxonomic identity at species level (necessitating confirmatory PCR) (2, 4, 8). In addition, these methods generally necessitate the application of analytic approaches using descriptive measures of richness, evenness and dominance which are more appropriate for samples of very much higher bacterial abundance than those observed in the paucibacillary asthmatic airway (13).

Identifying “treatable traits” (5), including type-2 cytokine-mediated (T2-high) eosinophilic airway inflammation, has enabled highly-effective biomarker-directed approaches, targeting novel biologics according to blood and sputum eosinophilia, and fractional exhaled nitric oxide (FeNO) (5). T2-low non-eosinophilic disease is refractory to corticosteroids and existing biologics (5) and remains poorly understood. It is present in up to 30% of severe asthmatics (14), associated with airways neutrophilia, high expression of IL-17 (14) and its inducible chemokines in airway T cells, and may be driven by chronic bacterial airways infection (8, 15, 16). The macrolide antibiotic azithromycin has emerged as a potential targeted therapy for this cohort (11, 16). However, new tools are required to accurately characterize the airway microbiome direct from patient samples, both for targeting of therapies on an individual patient basis, and to provide insight into disease mechanisms.

We hypothesize that chronic bacterial airways infection is a key treatable trait, however its prevalence, clinical phenotype, and biomarkers are yet to be defined. Comprehensive coverage of airway microorganisms to a species level has not been reported independently with metagenomic sequencing in severe asthma. This approach could define the prevalence of airways infection in severe asthma and identify the most relevant pathogenic species in an unbiased manner, thereby facilitating biomarker validation and further characterization of the clinical phenotype when applied to a large cohort. In this pilot study we have optimized a method of metagenomic sequencing of total DNA extracts from induced sputum samples collected from the Oxford Severe Asthma Cohort during clinical stability using the Oxford Nanopore Technologies (ONT) platform. Oxford Nanopore sequencing has the advantages of rapid and lower cost library preparation and sequencing, real-time data acquisition and longer contiguous reads offering improved *de novo* genome reconstruction. We compared Oxford Nanopore sequencing with previously utilized short-read Illumina MiSeq sequencing alongside pathogen specific quantitative PCR assays and “gold standard” sputum culture to test the validity of this method and identified a high prevalence of Haemophilus influenzae associated with neutrophilic disease in stable severe asthma.

## RESULTS

### Overview of airway microbiome in severe asthma.

**FIG 1:**
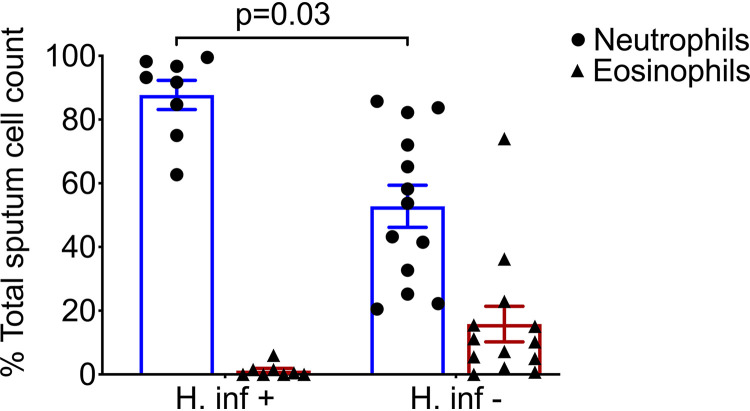
Presence of H. influenzae is associated with sputum neutrophilia; sputum differential cell counts stratified by presence or absence of *H. influenzae* by Oxford Nanopore sequencing and quantitative PCR. Statistical significance of eosinophil: neutrophil ratio assessed by Mann-Whitney test (*P* = 0.03).

Induced sputum samples were obtained during clinical stability from 23 participants with severe asthma. Clinical characteristics are shown in Table 1 and further summarized by absence (HI–) or presence (HI+) of H. influenzae, which emerged as the dominant bacterial species most frequently detected using culture based and molecular techniques in this study. Demographic data of the healthy participant are provided in Table S1 in the supplemental material. In this small cohort, 52% of asthmatic individuals were ex-smokers with a mean history of 12 pack years, and no difference in smoking history observed between the HI– and HI+ groups. All participants with severe asthma were established on high dose inhaled corticosteroids and 39% on maintenance oral corticosteroids. Blood eosinophilia (>0.3 × 10^9^/L) was seen in a greater proportion of HI– (11/15, 73%) compared with HI+ (4/8, 50%) participants. Presence of H. influenzae in culture, or positive pathogen specific PCR assay (described below), was associated with sputum neutrophilia and a significantly lower ratio of sputum eosinophils:neutrophils (*P* = 0.03, Mann-Whitney test, Fig. 1) with relatively lower FeNO levels; consistent with a type 2 biomarker-low phenotype (17).

**TABLE 1:** Clinical characteristics and airway inflammatory cell parameters of participants according to presence of airway H. influenzae^*a*^

Characteristic	Severe asthma (*n* = 23)	H. influenzae negative (*n* = 15)	H. influenzae positive (*n* = 8)	*P* value
Male sex, *n* (%)	13 (57)	9 (69)	4 (31)	0.66
Age (yr), mean (SD)	67 (10)	69 (9)	62 (10)	0.07
BMI (kg/m^2^), mean (SD)	28.4 (6.10)	28.2 (6.20)	28.8 (28.2)	0.83
Presence of atopy, *n* (%)	11 (48)	6 (55)	5 (46)	0.33
Presence of nasal polyps, *n* (%)	7 (30)	4 (57)	3 (43)	0.61
Ex-smoker, *n* (%)	12 (52)	7 (58)	5 (42)	0.49
Pack yr, mean (SD)	12 (10)	11 (6)	14 (15)	0.62
Baseline inhaled corticosteroid use (BDP eq., mcg/d), median (Range)	2,000 (1,700)	2,000 (1,700)	2,000 (1,200)	0.97^*b*^
Maintenance oral corticosteroid, *n* (%)	9 (39)	8 (89)	1 (11)	0.06
Unscheduled GP/hospital visits in 12 mo, median (range)	4 (7)	4 (5)	5 (6)	0.79^*b*^
Corticosteroid courses in 12 mo, median (range)	3 (10)	2 (10)	3 (9)	0.84^*b*^
Antibiotic courses in 12 mo, median (range)	0 (7)	0 (7)	2 (6)	0.4^*b*^
FEV1 (% predicted), mean (SD)	69.6 (16.8)	69.9 (15.1)	68.9 (20.8)	0.58^*b*^
FEV1/FVC, mean (SD)	0.58 (0.10)	0.56 (0.10)	0.62 (0.10)	0.21
FeNO (ppb), mean (SD)	62 (68)	75 (74)	40 (53)	0.26
Blood eosinophils (×10^9^/L), mean (SD)	0.50 (0.44)	0.53 (0.35)	0.45 (0.58)	0.56^*b*^
Sputum eosinophils (%), median (range)	0.50 (23)	1 (23)	0.10 (2)	**0.01** ^*b*^
Sputum neutrophils (%), median (range)	72 (79)	54 (65)	93 (37)	**<0.01** ^*b*^
Sputum inflammatory phenotype, *n* (%)				
Total with valid data	21	13	8	
Eosinophilic	5 (24)	5 (38)	0 (0)	
Neutrophilic	13 (62)	5 (38)	8 (0)	
Mixed granulocytic	0 (0)	0 (0)	0 (0)	
Paucigranulocytic	3 (14)	3 (23)	0 (0)	

a BMI, body mass index; GP, general practitioner; FEV1, forced expiratory volume in 1s; FVC, forced vital capacity; FeNO, exhaled nitric oxide; 1. Percentages given are provided from subjects with valid daata; Inflammatory phenotyes: eosinophilic >3% sputum eosinophils, neutrophilic >61% sputum neutrophils and <3% eosinophils, mixed granulocytic >61% sputum neutrophils and >3% eosinophils, paucigranulocytic <61% sputum neutrophils and <3% eosinophils.

b Comparisons between groups made using unpaired *t* test or Mann-Whitney test. *P* values < 0.05 are highlighted in bold.

### DNA extraction and performance of sequencing.

DNA was extracted from induced sputum plug samples using the QIAamp DNA minikit with prior human DNA depletion using differential centrifugation. Differential centrifugation produced 2.6 to 2.7-fold increase in total unfiltered bacterial reads compared with a 2.9 to 3.1-fold increase in bacterial reads with the NEBNext Microbiome kit, this corresponded to up to 8.2% and 13.5% reduction in proportion of unfiltered human reads respectively (Fig. S1). Differential centrifugation was selected as it provided additional benefits of ease of sample processing and lower cost per sample.

A pilot method was tested to compare gold standard sputum culture with metagenomic sequencing on the Illumina MiSeq and Oxford Nanopore platforms with quantification of bacterial load using qPCR performed in parallel. Taking the approach of metagenomic sequencing of total DNA transcripts with Illumina MiSeq and Oxford Nanopore over 16S rRNA gene amplicon sequencing identified dominant airway organisms to a species (over genus) level. All organisms identified through standard sputum culture are shown in Table S2. H. influenzae was the most common pathogenic organism within the airway in severe asthma (*n* = 7/23) and positive culture was predictive of dominance within the airway metagenome defined as the majority of total bacterial reads and bacterial bases occupied by a single species (Table 2). In addition, total H. influenzae load was quantified by qPCR using >1 × 10^6^ copies/mL as a cutoff for clinically significant infection as previously validated in airways infection (18) (*n* = 8/23)—this assay yielded a positive result in one participant with severe asthma in the context of a negative sputum culture (Table 2).

**TABLE 2:** Summary of sputum microbiology; pilot data (*n* = 23), sputum differential (neutrophilia >61%, eosinophilia >3%), FeNO (high: >45 ppb), culture, MALDI-TOF speciation, DNA sequencing by Oxford Nanopore; RT-qPCR for Eubacteria 16S and specific pathogens: *H. influenzae*, *S. pneumoniae*, *M. catarrhalis*, *P. aeruginosa*, *S. aureus* (positive >1 × 10^6^ copies/mL)^*a*^

PID	Age (yrs)	Sex	Smoking status (pack yrs)	*H. inf* culture	*H. inf* reads (% total bacterial reads)	H. inf qPCR (×10^6^ copies/mL)	S. pneu qPCR (×10^6^ copies/mL)	M. cat qPCR (×10^6^ copies/mL)	P. aeru qPCR (×10^6^ copies/mL)	S. aur qPCR (×10^6^ copies/mL)	Total 16S qPCR (×10^6^ copies/mL)	Sputum neutrophils (%)	Sputum eosinophils (%)	FeNO (ppb)
1054	53	M	Ex (10)	+	93.5	**983.80**	**1.89**	−	−	−	8510	**93.2**	0.5	nd
278	50	M	Ex (40)	+	86.5	**435.40**	−	0.79	−	−	2832	**84.7**	0	11
133	60	F	Never	+	66.6	**91.70**	**1.99**	−	−	−	1643	**98.2**	0	29
297	76	F	Never	+	66.6	**40.00**	**1.72**	−	−	−	1042	**75**	0	21
288	67	M	Ex (9)	+	58.0	**31.00**	−	−	−	0.02	572	**96.7**	0.2	**169**
29	76	F	Ex (10)	+	47.1	**16.50**	0.17	−	−	−	633	**62.7**	0.5	9
214	54	M	Ex (<1)	+	17.1	**12.50**	−	−	−	−	338	**99.5**	0	20
1049	57	F	Never	−	51.3	**12.40**	−	−	−	−	297	**91.7**	2	20
235	57	M	Never	−	3.3	0.70	−	−	−	−	335	20.5	0.5	37
306	69	F	Never	−	0.03	0.30	−	−	−	−	1006	**82.2**	1	**85**
187	66	M	Ex (12)	−	0.05	0.03	−	−	−	−	1822	25.2	**7.5**	**300**
1052	79	M	Never	−	0.1	0.02	−	**35.63**	−	−	695	**65.2**	1.2	**125**
294	62	F	Never	−	0.05	0.02	−	−	−	−	43	nd	0.5	28
1055	70	F	Ex (15)	−	0.6	0.01	−	−	−	−	91	58.2	**15.5**	**138**
1053	59	M	Ex (10)	−	0.5	0.01	−	−	−	−	1754	**85.7**	0.7	**64**
1041	73	F	Ex (8)	−	1.0	0.01	−	−	−	−	286	43.2	0.5	8
5	67	M	Never	−	0.07	−	−	−	−	−	661	53.7	11	8
275	76	F	Never	−	2.8	−	**5.95**	−	−	−	696	**83.7**	0.7	20
315	61	M	Never	−	0.05	−	−	−	−	−	115	22.2	0.2	**78**
1060	86	M	Ex (8)	−	0.0	−	−	−	−	−	58	32.7	**22.9**	16
279	60	F	Never	−	0.05	−	−	−	−	−	216	nd	nd	48
295	81	M	Ex (20)	−	0.4	−	−	−	−	−	191	**72**	0	**78**
1058	72	M	Ex (3)	−	1.0	−	−	−	−	−	95	41.5	**15.1**	**89**

a Values above indicated thresholds levels shown in bold denotes pathogen not detected by PCR. Participants are ranked according to detection of *H. influenae* by quantitative PCR. +, denotes pathogen detected; −, denotes pathogen not detected by standard culture or by PCR.

**FIG 2:**
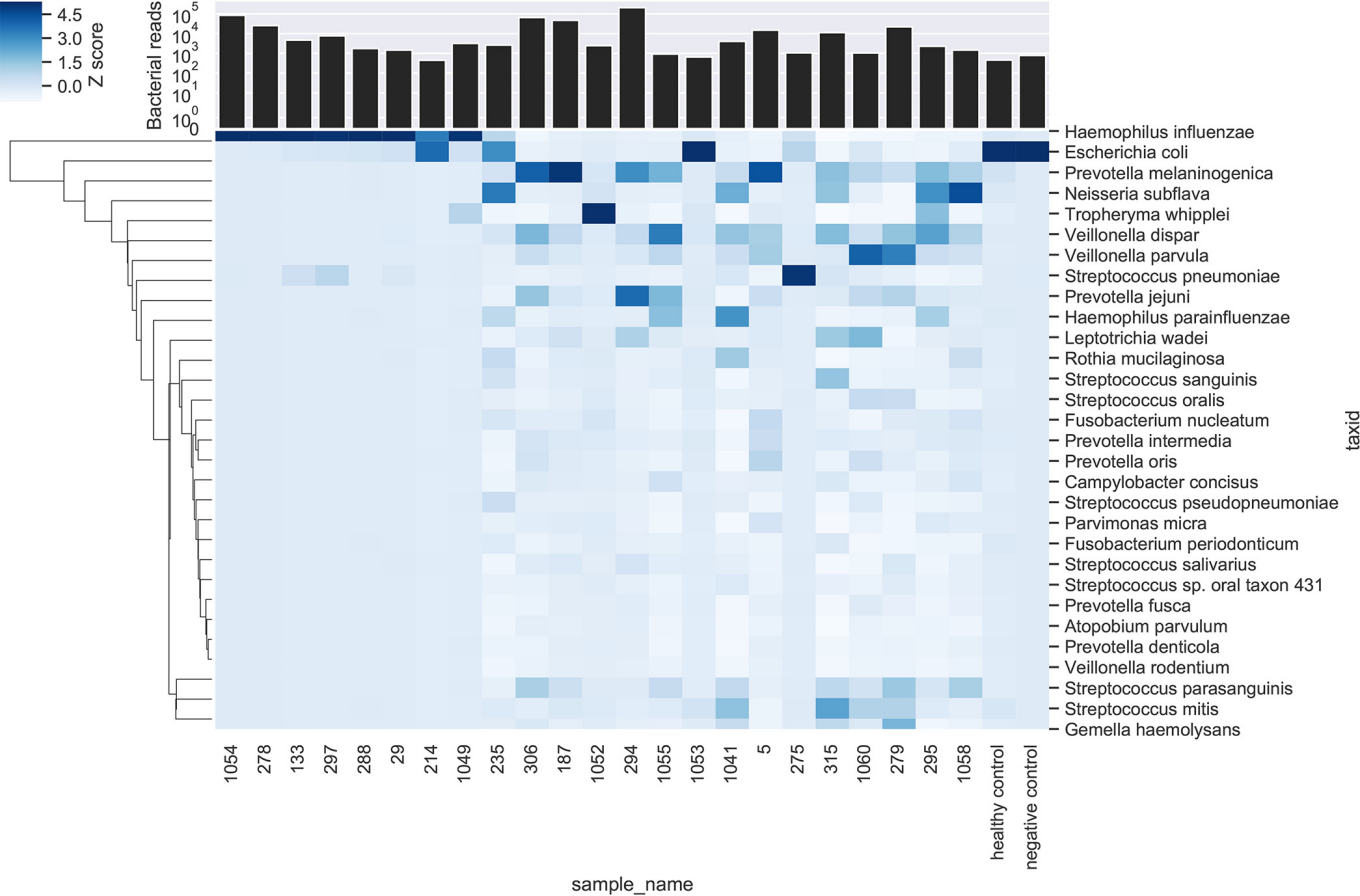
Heatmap of relative abundance of species sequenced by sample using ONT; Z-scores, denoted by shade, represent the number of standard deviations above the mean number of bases per taxon for each sample. Total bacterial reads detected per sample are also shown in stacked bar chart.

**FIG 3:**
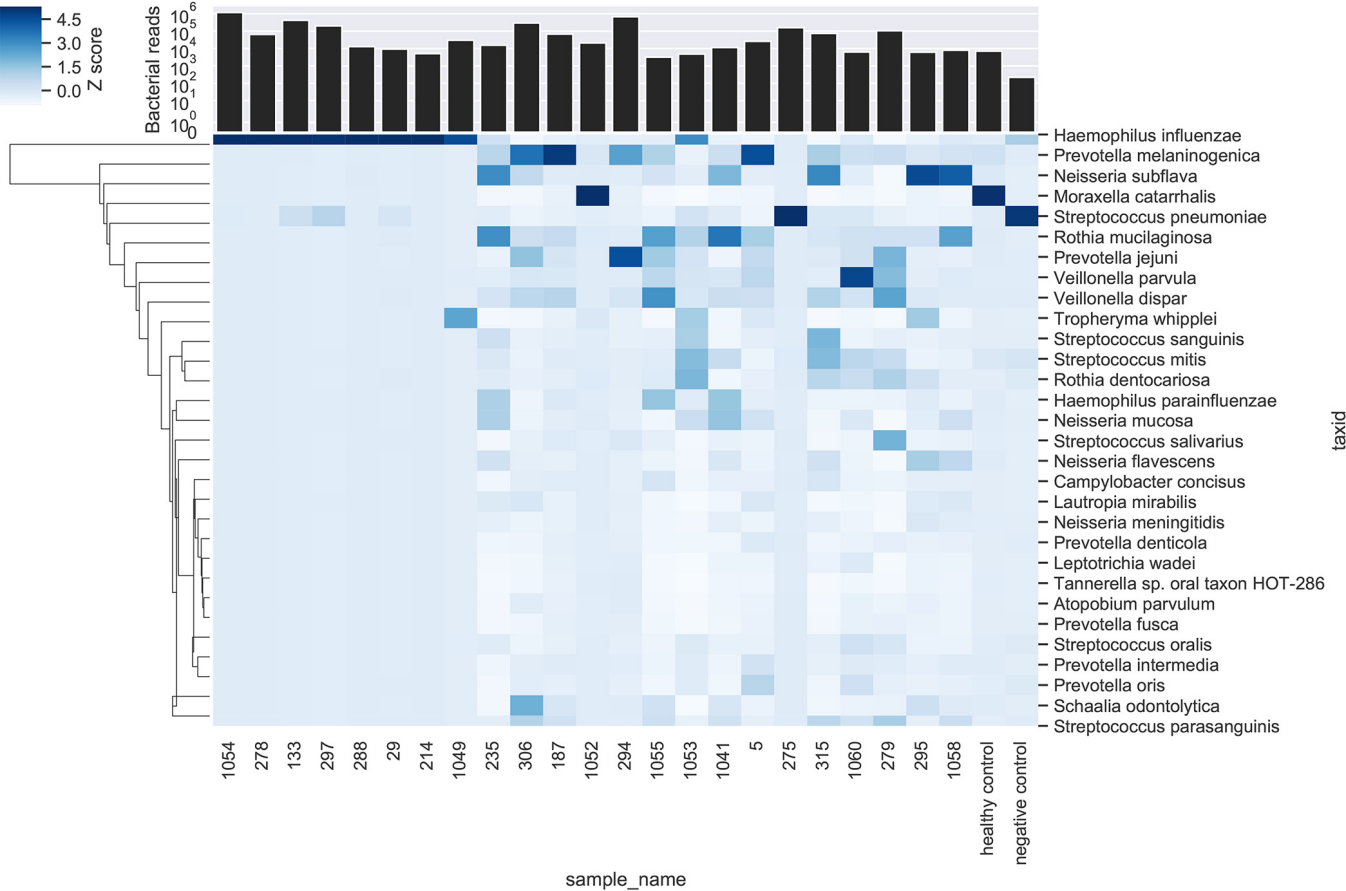
Heatmap of relative abundance of species sequenced by sample using Illumina MiSeq; Z-scores, denoted by shade, represent the number of standard deviations above the mean number of bases per taxon for each sample. Total bacterial reads detected per sample are also shown in stacked bar chart.

**FIG 4:**
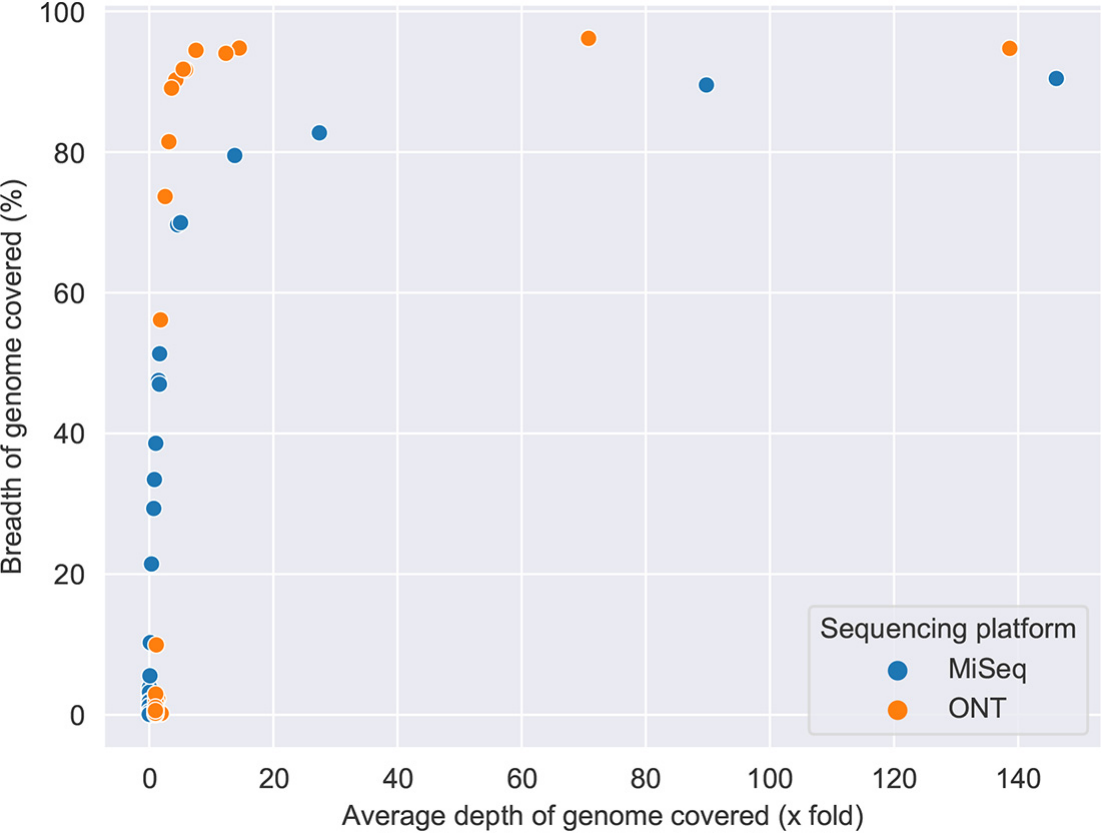
Comparison of read lengths and genome coverage for H. influenzae using Oxford Nanopore compared with Illumina MiSeq; breadth and depth of H. influenzae genome coverage with Oxford Nanopore and Illumina MiSeq in all samples.

**FIG 5:**
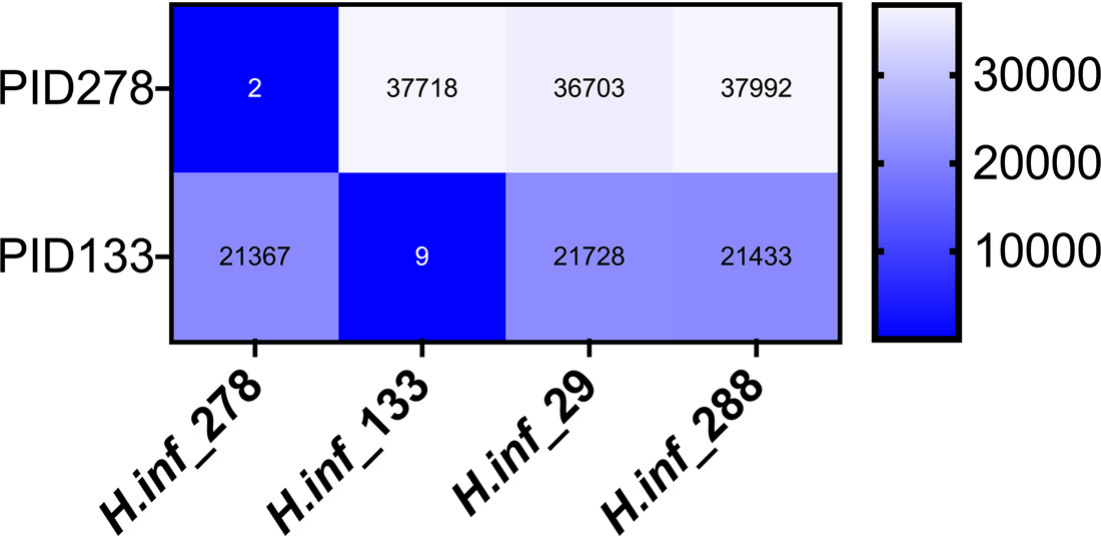
SNP distance matrix; pairwise comparisons between metagenomic sequences from clinical samples and sequenced clinical H. influenzae isolates from 4 patients following Oxford Nanopore sequencing are shown.

A head-to-head comparison was made of Oxford Nanopore and Illumina MiSeq. Total human and bacterial reads detected by metagenomic sequencing using Oxford Nanopore and Illumina MiSeq are shown in Fig. S2 in the supplemental material, alongside the number of human reads removed bioinformatically prior to downstream analysis. Raw read statistics from fastq files have been generated using SeqKit (19) and summarized in Supplemental data file 1. Overall, this demonstrates longer reads lengths achieved with Oxford Nanopore (median of average read length/sample: Oxford Nanopore 2,013 bp versus Illumina MiSeq 172 bp) and higher quality scores using Illumina MiSeq (median [IQR] Q20: Oxford Nanopore 50% [41%] versus Illumina MiSeq 76% [20%]). Consistent taxonomic agreement was seen between these technologies. Similar microbiota compositions were observed when species level taxonomy was compared, as shown in Table 3 using paired HI+ samples, and heatmaps (Fig. 2 and 3) showing relative abundance of species detected in all samples. A difference is noted in the negative control sample when analyzed by Ilumina MiSeq and Oxford Nanopore, with the classification of a small number of reads to Escherichia coli with Oxford Nanopore. This is likely to represent low level contamination or metagenomic misclassification, thereby also accounting for the abundance of E. coli in samples with relatively low total reads (participant identifier [PID]: 214, 235, 1053, 275). Metagenomic sequencing of the negative control sample using Illumina MiSeq detected 1100 bacterial reads (median bacterial reads in clinical samples was 22,563), of which S. pneumoniae occupied 12% of bacterial reads (135 reads). The negative control sample fell below the limit of detection of the S. pneumoniae PCR assay and is not considered a significant contaminating signal as it has not appeared as a dominant organism in other samples with a negative PCR result, where the proportion of reads are comparable to Oxford Nanopore (Table 3), and specifically S. pneumoniae occupied 88% of reads (149,030/168,844 bacterial reads) in PID 275 where a clearly positive PCR result is seen. Using Oxford Nanopore at least 88.9% of the reference H. influenzae genome was covered by ≥1 read in most (*n* = 7/8) HI+ samples, with only one sample achieving <25% coverage breadth (Table 4). In addition, compared with Illumina MiSeq, Oxford Nanopore provided greater H. influenzae genome coverage at any given depth of sequencing compared with Illumina MiSeq in all sequenced samples (Fig. 4). To study whether Oxford Nanopore can discriminate between H. influenzae strains, 4 strains isolated from clinical samples (PID 278,133,29 and 288) were cultured and sequenced. Consensus sequences were generated for cultured H. influenzae and their corresponding clinical samples (Table S3). The consensus sequences of two clinical samples (PID 278 and 133) achieved adequate coverage (91.4% and 53.8%, respectively) for reliable comparisons to be made with cultured strains. As shown in the single nucleotide polymorphism (SNP) distance matrix in Fig. 5, there is a high degree of similarity (very low SNPs) between clinical samples and the corresponding strain of H. influenzae isolated.

**TABLE 3:** Agreement between Oxford Nanopore and Illumina MiSeq in HI+ samples; displayed in “top 5” bacterial species occupying the majority of total bacterial reads per sample (*n* = 8)

PID	Oxford Nanopore technologies	% Bacterial reads	Ilumina MiSeq	% Bacterial reads
1054	Haemophilus influenzae	93.5	Haemophilus influenzae	90.9
	Streptococcus pneumoniae	0.8	Streptococcus pneumoniae	1.3
	Escherichia coli	0.3	Neisseria meningitidis	0.4
	Neisseria meningitidis	0.3	Tropheryma whipplei	0.3
	Haemophilus haemolyticus	0.2	Haemophilus haemolyticus	0.1
278	Haemophilus influenzae	86.5	Haemophilus influenzae	82.6
	Escherichia coli	1.3	Moraxella catarrhalis	0.1
	Salmonella enterica	0.3	Haemophilus parainfluenzae	0.1
	Shigella flexneri	0.2	Haemophilus haemolyticus	0.1
	Helicobacter pylori	0.2	Streptococcus pseudopneumoniae	0.1
133	Haemophilus influenzae	66.6	Haemophilus influenzae	72.9
	Streptococcus pneumoniae	7.3	Streptococcus pneumoniae	8.8
	Escherichia coli	2.7	Haemophilus haemolyticus	0.6
	Salmonella enterica	0.6	Prevotella melaninogenica	0.2
	Shigella boydii	0.5	Haemophilus parainfluenzae	0.2
297	Haemophilus influenzae	66.6	Haemophilus influenzae	67.6
	Streptococcus pneumoniae	12.8	Streptococcus pneumoniae	14.4
	Escherichia coli	3.2	Haemophilus parainfluenzae	0.1
	Shigella boydii	0.4	Haemophilus haemolyticus	0.1
	Salmonella enterica	0.3	Streptococcus mitis	0.1
288	Haemophilus influenzae	58.0	Haemophilus influenzae	79.8
	Escherichia coli	3.7	Neisseria subflava	0.9
	Shigella flexneri	1.0	Haemophilus aegyptius	0.5
	Salmonella enterica	0.9	Haemophilus parainfluenzae	0.5
	Shigella boydii	0.7	*[* Haemophilus *] ducreyi*	0.4
29	Haemophilus influenzae	47.1	Haemophilus influenzae	69.4
	Escherichia coli	4.6	Streptococcus pneumoniae	4.6
	Streptococcus pneumoniae	1.8	Rothia dentocariosa	1.0
	Shigella boydii	0.9	Rothia mucilaginosa	1.0
	Salmonella enterica	0.9	Veillonella dispar	0.8
214	Haemophilus influenzae	17.1	Haemophilus influenzae	80.7
	Escherichia coli	15.4	Haemophilus haemolyticus	0.3
	Helicobacter pylori	3.6	Prevotella oris	0.3
	Salmonella enterica	1.9	Veillonella dispar	0.2
	Shigella flexneri	1.3	Haemophilus parainfluenzae	0.2
1049	Haemophilus influenzae	51.3	Haemophilus influenzae	53.7
	Tropheryma whipplei	10.9	Tropheryma whipplei	29.9
	Escherichia coli	5.2	Veillonella parvula	0.6
	Shigella boydii	0.8	Haemophilus haemolyticus	0.3
	Shigella flexneri	0.6	Streptococcus pseudopneumoniae	0.2

**TABLE 4:** H. influenzae genome coverage breadth and depth with Oxford Nanopore; total and *H. inf* specific reads and bases in HI+ samples shown with coverage breadth (percentage of reference sequences covered), average coverage depth (at positions with ≥1 read) and number of positions on the H. influenzae genome with 1/5/10 read depth

PID	Bacterial reads	*H. inf* reads	*% H. inf*/bacterial reads	Bacterial bases	*H. inf* bases	*% H. inf/bacterial bases*	Legth of *H. inf* reference genome (bases)	Coverage breadth *H. inf* genome	Avg coverage depth	*H. inf* genome positions with:		
										1 read depth	5 reads depth	10 reads depth
1054	88101	82374	93.5	4.97E + 08	4.85E + 08	97.7	1.83E + 06	94.2	243.3	1.72E + 06	1.71E + 06	1.70E + 06
278	28460	24609	86.5	1.14E + 08	1.12E + 08	97.9	1.83E + 06	93.4	53.6	1.71E + 06	1.70E + 06	1.69E + 06
133	6054	4031	66.6	2.43E + 07	2.16E + 07	89.1	1.83E + 06	92.7	10.1	1.70E + 06	1.63E + 06	7.48E + 05
297	9228	6142	66.6	4.32E + 07	3.52E + 07	81.6	1.83E + 06	92.9	17.5	1.70E + 06	1.69E + 06	1.60E + 06
288	2775	1610	58.0	9.06E + 06	8.47E + 06	93.5	1.83E + 06	90.7	4.2	1.66E + 06	6.80E + 05	9.49E + 03
29	2604	1226	47.1	6.97E + 06	5.85E + 06	83.8	1.83E + 06	88.9	3.2	1.63E + 06	3.36E + 05	1.06E + 02
214	1344	230	17.1	8.53E + 05	6.68E + 05	78.4	1.83E + 06	24.2	1.3	4.43E + 05	0.00E + 00	0.00E + 00
1049	4632	2376	51.3	1.62E + 07	1.21E + 07	74.5	1.83E + 06	92.1	6.1	1.69E + 06	1.21E + 06	1.04E + 05

### Confirming presence of other coexistent respiratory pathogens.

The PCR panel was extended to include Streptococcus pneumoniae, Staphylococcus aureus, Moraxella catarrhalis and Pseudomonas aeruginosa in addition to H. influenzae (Table 2). This identified presence of S. pneumoniae alongside H. influenzae above a threshold of >1 × 10^6^ copies/mL—previously shown to be clinically significant (18)—in a proportion of participants (*n* = 3). S. pneumoniae constituted the second highest proportion of bacterial reads within these samples and was identified on sputum culture. In this small cohort a positive PCR result for M. catarrhalis and S. pneumoniae alone was detected in one participant each and associated with a sputum neutrophilia. S. pneumoniae was the dominant organism on sequencing with Oxford Nanopore (PID: 275) in this case. M. catarrhalis was detected as abundant (74.5% total bacterial reads) by Illumina MiSeq, but not by Nanopore (<1% total bacterial reads) in one individual (PID: 1052) in whom a positive PCR result was present, but sputum culture was negative (Table 2 and Fig. 3). M. catarrhalis was detected by Oxford Nanopore in PID 278, albeit at low levels (39/28,460 total bacterial reads, 0.1% of bacterial reads) in the context of dominance of H. influenzae in this sample and M. catarrhalis PCR result falling just below the threshold of clinical significance (0.86 × 10^6^ copies/mL).

### Limits of detection and spiking.

**FIG 6:**
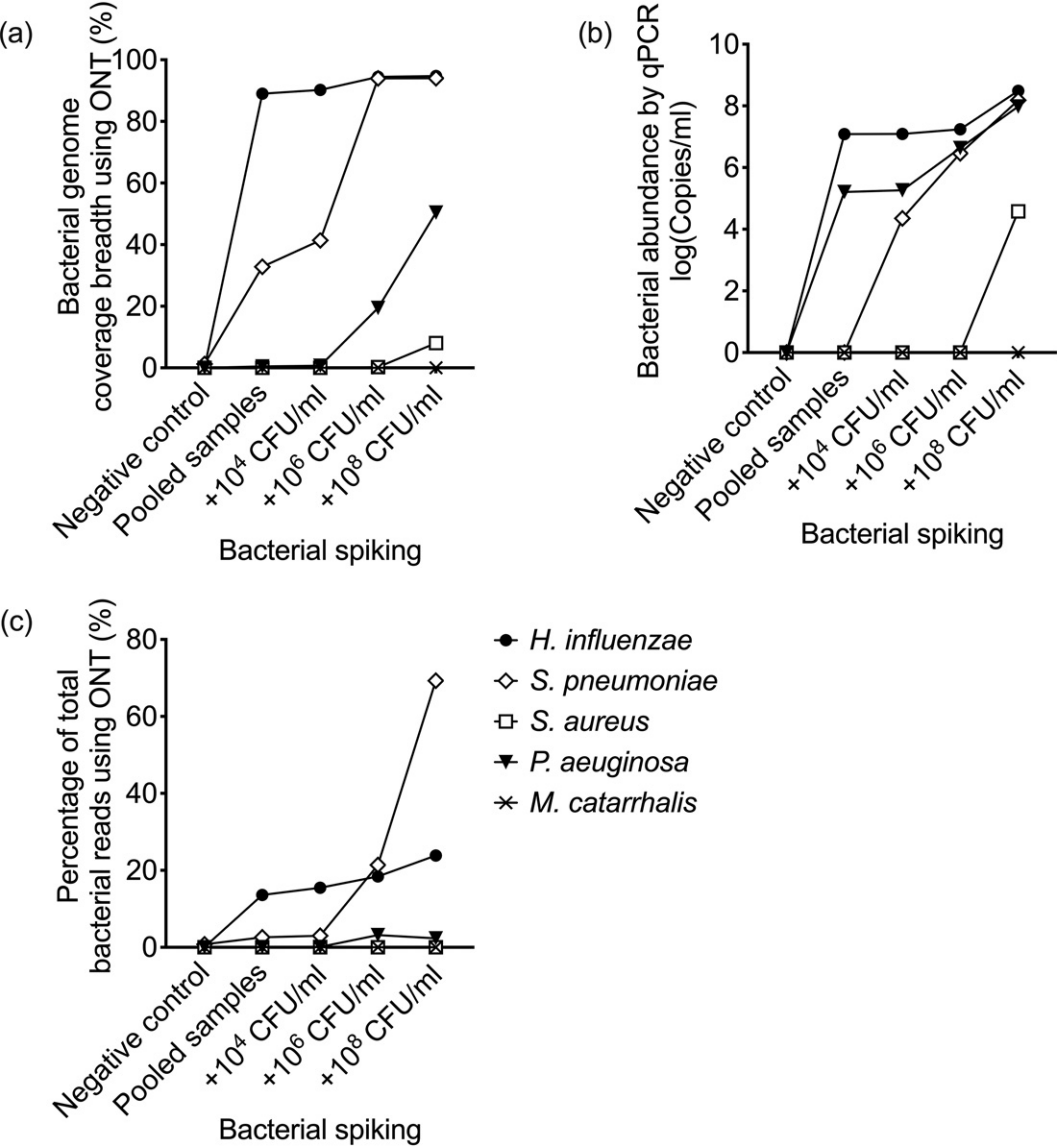
Spiking recovery in sputum samples by Oxford Nanopore (ONT) and by qPCR. Recovery of spiked organisms (H. influenzae, S. pneumoniae, S. aureus, P. aeruginosa, M. Catarrhalis) on metagenomic sequencing using Oxford Nanopore, expressed as (a) % target bacterial genome coverage breadth and (c) % bacterial reads; or (b) detected by pathogen specific PCR.

A pooled sample was generated from individuals with no single dominant airway organism to capture the variation in lung microbiota within a cohort of severe asthmatics during stable disease. Spiking with 10^4^ to 10^8^ CFU/mL of Haemophilus influenzae, Streptococcus pneumoniae, Staphylococcus aureus, Moraxella catarrhalis, and Pseudomonas aeruginosa was performed. There was an increase in total number of bacterial reads with increasing concentration of bacterial spike which corresponded to rising bacterial load from a high background level of 4.8 × 10^8^ copies/mL (in the unspiked pool) to 1.5 × 10^10^ copies/mL at the highest spiking concentration. Reads classified as Haemophilus influenzae, Streptococcus pneumoniae, Staphylococcus aureus, Moraxella catarrhalis and Pseudomonas aeruginosa were observed in the pooled un-spiked sample and spiked samples. H. influenzae and S. pneumoniae demonstrated the greatest rise in proportion of assigned bacterial reads with spiking as shown in Fig. 6c. Near complete genome coverage of these organisms was achieved after spiking with 10^6^ CFU/mL, followed by 50% genome coverage of P. aeruginosa at this concentration despite only a modest increase in bacterial reads (Fig. 6a). Recovery of S. aureus and M. catarrhalis following spiking was poor by Oxford Nanopore or by qPCR (Fig. 6). As shown in Fig. 6b there was a high background level of airway H. influenzae (>1 × 10^6^ copies/mL) within the pooled sample, crossing the threshold of clinical significance. This threshold was surpassed by S. pneumoniae and P. aeruginosa at spiking concentrations exceeding 10^6^ CFU/mL but could not be achieved upon spiking S. aureus or M. catarrhalis up to concentrations of 10^8^ CFU/mL.

## DISCUSSION

In this study we report for the first time a method for DNA extraction and direct metagenomic sequencing of induced sputum samples using Oxford Nanopore in stable severe asthma. In severe asthmatics demonstrating altered microbial diversity, marked by the dominance of a single pathogenic bacterial species in the airway, H. influenzae was the most prevalent organism. These results were validated using the established short read sequencing platform Illumina MiSeq, quantitative PCR and “gold standard” sputum culture. Oxford Nanopore has previously been used for diagnosis of bacterial lower respiratory tract infection (20) with higher biomass than respiratory samples utilized in our cohort of stable severe asthmatics. Despite this Oxford Nanopore achieved near complete bacterial genome coverage in the majority of HI+ samples.

The selected method of DNA extraction with preceding differential centrifugation provided 2.6- to 2.7-fold increase in bacterial reads. Previous studies have more extensively compared different techniques of human/extracellular DNA depletion from sputum samples; either from patients with cystic fibrosis (21) or bronchiectasis (22) using metagenomic or 16S sequencing on the Illumina MiSeq platform or from complex airways samples isolated at the time of pulmonary infection for metagenomic sequencing using Oxford Nanopore (20). These respiratory samples are distinct from sputum plug samples utilized in this study, collected during stable disease from patients with severe asthma with no concurrent antimicrobial therapy or clinical diagnosis of bronchiectasis. Use of differential centrifugation yielded a modest reduction in human DNA but allowed consistent species level discrimination with robust bacterial genome coverage in airways infection. More complete human DNA depletion may be possible to achieve, without loss of bacterial genomes, but was not within the scope of this study. When performing Oxford Nanopore sequencing, six samples were run per flow cell. This produced adequate sequencing depth and breadth for species level taxonomic identification, but potentially higher sequencing yield could be achieved, if required, by reducing the number of multiplexed samples although this would have implications on cost per sample. Such an approach may be favorable for making more detailed inferences about dominant bacterial species directly from metagenomic sequencing data as we have shown that clinical samples with high level consensus genome coverage have few SNPs when compared to direct sequencing of corresponding cultured strains of H. influenzae. The presence of a limited number of SNPs seen in this study between metagenomic analysis of clinical samples and paired sequences of cultured bacterial strains could be explained by the high level of genomic diversity seen in H. influenzae (23), particularly within non-capsulate strains most often isolated in airways disease (24–26). This method is a significant step forward from 16s rRNA sequencing by identifying dominant species clearly as those occupying the highest proportion of total bacteria reads and bases.

Oxford Nanopore was superior to Illumina MiSeq within this cohort in providing greater breadth of bacterial genome coverage within the HI+ group at any sequencing depth. Oxford Nanopore has a relatively high per base error rate of up to 6%, necessitating bioinformatic approaches to overcome this in base-called sequences and produce an acceptable consensus genome (27). Randomly generated sequencing errors can be compensated for by achieving adequate sequencing depth (28). In our data set there is a high level of agreement between Oxford Nanopore and Illumina MiSeq, reported to have an error rate <0.5% (29), as exemplified in Table 3. This is most notable in samples with a positive quantitative pathogen PCR result or culture. An exception to this was noted in a single sample (PID: 1052) in which only Illumina MiSeq detected the presence of M. catarrhalis in the presence of a positive PCR result. This is likely an artifact of Oxford Nanopore library preparation rather than a systematic sequencing technology error. In ongoing work within a larger cohort, Oxford Nanopore sequencing has identified M. catarrhalis as a dominant organism reliably on metagenomic sequencing with concordant positive PCR results (data not shown). Spiking experiments have shown poor recovery of M. catarrhalis up to spiking concentrations of 10^8^ CFU/mL, however there was high background bacterial DNA levels in these samples.

Our study has several limitations. Firstly, a small sample size has been used for method development. This study lacks adequate statistical power to define cutoffs based on sequencing metrics, such as proportion of bacterial reads or bacterial bases, to support a clinically relevant diagnosis of airways infection. Further prospective validation is required in larger patient cohorts and is under way. Secondly there was additional value in performing pathogen specific PCR given the variation in limits of detection of bacterial species by sequencing within our spiking experiment. A high background bacterial level in the un-spiked pooled sputum samples restricted our ability to compare limits of detection between qPCR and Oxford Nanopore, although our data suggested both were sensitive for H. influenzae, with superior sensitivity of qPCR for P. aeruginosa and of Oxford Nanopore for S. pneumoniae, suggesting operational characteristics may differ between technologies according to individual species. Additionally our study did not integrate metagenomic data with functional immunological readouts, an area warranting further study in an adequately powered cohort of well-phenotyped patients.

H. influenzae is emerging as the most relevant potentially pathogenic organism in severe asthma (2, 7, 8), however its overall prevalence is yet to be defined alongside a full characterization of clinical phenotype and longitudinal disease outcomes. Abundance of H. influenzae is predictive of response to treatment with the macrolide antibiotic azithromycin: the only established targeted therapy in T2-low asthma. This highlights the importance of identifying candidate bacterial *species* as treatable traits (5) when characterizing disease phenotype over description of compositional changes of “airway dysbiosis.” The frequency of other common respiratory pathogens such as Streptococcus pneumoniae and Moraxella catarrhalis are not known in severe asthma. Colonization with these organisms alongside H. influenzae is predictive of wheeze and asthma in early life (30) and likely to be relevant independently or within polymicrobial communities through mechanisms including interspecies quorum sensing (31) in shaping the virulence of constituent bacteria.

In summary, we have optimized a method of direct metagenomic sequencing of induced sputum samples. Using this method, H. influenzae was reliably identified as a clinically-relevant pathogen in severe asthma. Future large cohort studies will be required to characterize the airway microbiome in specific asthma phenotypes, to determine the role of these other potentially pathogenic bacteria, and to explore the relationships between the presence of individual airway bacteria and the activation of specific immunological pathways which contribute to the poorly understood phenotypes of ‘T2 low’ asthma.

## MATERIALS AND METHODS

### Clinical Samples.

Patients meeting the American Thoracic Society/European Respiratory Society definition of severe asthma, on Global Initiative for Asthma (GINA) step 4–5 treatment (32) (*n* = 23) and healthy control (*n* = 1) were recruited to the Oxford Severe Asthma Cohort from the Churchill Hospital, Oxford, UK between December 2017 and June 2018. This study was conducted with NHS Research Ethics approval (08/H0406/189). Induced sputum samples were collected prospectively during clinical stability. Hypertonic sputum induction and sputum differential cell counts were performed as previously described (33) and sputum plugs stored at −80°C in sterile Brain Heart Infusion (BHI, Sigma-Aldrich, Dorset, UK) broth containing 10% glycerol until required. Sputum plugs were dispersed with 0.1% dithiothreitol (DTT, Thermo Fischer, Waltham, MA, USA) and filtered before microbiological culture and DNA extraction as described below.

### Sputum Culture.

Routine sputum culture was performed using homogenized sputum according to the Health Protection Agency (HPA) standard operating procedures. All cultured organisms were identified by matrix-assisted laser desorption/ionization time-of-flight (MALDI-TOF) mass spectrometry on a Microflex LT using Biotyper, version 3.1 (Bruker Daltonics, Ballerica, MA, USA). In addition, prospective Haemophilus species were also identified using X (hemin), V (NAD) and XV disks (hemin and NAD) (BD BBL Taxo differentiation discs for Haemophilus spp., Wokingham, UK) on nutrient agar (Oxoid Ltd, Basingstoke, UK). H. influenzae strains isolated from 4 clinical samples (PID 278, 133, 29 and 288) were cultured overnight (37°C with 5% CO2, shaking at 180 rpm) in 10 mL culture broth (BHI with 10 μg/mL NAD [NAD] and hemin [Sigma-Aldrich, Dorset, UK]). Once optical density at 600 nm reached 0.5 (exponential phase) bacteria were pelleted and resuspended in BHI containing 10% glycerol and stored at −80°C until use. Clinical laboratory results from the John Radcliffe Hospital Microbiology Laboratory, Oxford, UK have been provided for three samples.

### Microbial DNA extraction with human DNA depletion.

Two methods for extraction of high-quality microbial DNA and depletion of human cells/DNA, were tested. The first was differential centrifugation, whereby homogenized sputum was centrifuged (400 g, 5 min) to deplete human cells. The supernatant was collected, split into smaller volume aliquots (up to 1 mL) and centrifuged again (13,000g, 5 min). This supernatant was discarded, pellets from multiple aliquots combined for DNA extraction using the QIAamp DNA minikit (Qiagen, Manchester, UK) – briefly, combined pellets were resuspended in 180 μL buffer ATL (Qiagen, Manchester, UK) before adding 20 μL proteinase K to each sample and incubating at 56°C for 1 h, vortexing the sample every 15 min. Subsequent steps were as per manufacturer’s instructions. The second method for host DNA depletion utilized the NEBNext Microbiome DNA Enrichment Kit (New England Biolabs, Ipswich, MA, USA) as per manufacturer’s instructions—this kit facilitates enrichment of microbial DNA by selectively binding and removing CpG-methylated human DNA.

Microbial DNA extraction from clinical H. influenzae isolates (*H. inf*_278, *H. inf*_133, *H. inf*_29, and *H. inf*_288) was performed from overnight culture (37°C with 5% CO2) on chocolate agar (Oxoid Ltd, Basingstoke, UK). Bacterial colonies were deposited directly into buffer ATL (Qiagen, Manchester, UK) using flat 5 μL loops and DNA extracted as described above.

### Bacterial spiking.

Sputum plugs collected from five donors with no significant growth of potentially pathogenic respiratory organisms on culture (PID: 1052, 5, 1055, 315, 187) were homogenized with DTT as above and pooled together to generate a baseline sample for all spiking conditions. The pooled sample was then split for spiking simultaneously with Haemophilus influenzae, Streptococcus pneumoniae, Staphylococcus aureus, Moraxella catarrhalis and Pseudomonas aeruginosa. Bacteria were cultured overnight (37°C with 5% CO_2_, shaking at 180 rpm) in 10 mL culture broth as indicated by species (H. influenzae in BHI with 10 μg/mL NAD and hemin [Sigma-Aldrich, Dorset, UK], S. pneumoniae in BHI and S. aureus*/*M. catarrhalis*/*P. aeruginosa in nutrient broth [Oxoid]). Once optical density at 600 nm reached 0.5 (exponential phase) bacteria were pelleted and resuspended in BHI or nutrient broth containing 10% glycerol and stored at −80°C until use. Serial dilutions (in phosphate-buffered saline) and confirmatory CFU counts were performed to determine bacterial stock concentrations. The five cultured bacterial species were spiked simultaneously from stock solutions into the pooled homogenized sputum samples, producing three spiked samples containing 10^4^, 10^6^ or 10^8^ CFU/mL of each bacterial species. Microbial DNA was extracted, as described previously, from the unspiked and spiked pooled samples.

### Metagenomic sequencing.

**(i) Illumina MiSeq**. After quantification with a Qubit 2.0 fluorometer (Life Technologies, Paisley, UK), DNA extracts were sequenced on a MiSeq desktop sequencer (Illumina, San Diego, CA, USA). Libraries were prepared using a variation of the Illumina Nextera XT protocol (34). Briefly, 1 ng of DNA was prepared using the Nextera XT protocol modified with 15 cycles during the index PCR. DNA quantification with a Qubit 2.0 fluorometer was followed by determination of their average sizes on an Agilent 2200 TapeStation (Agilent Technologies, Santa Clara, CA, USA) before samples were manually normalized. Paired-end sequencing was performed using a 600-cycle MiSeq reagent kit (version 3), and samples were sequenced in batches of 12 on a single flow cell.

**(ii) Oxford Nanopore Technologies**. Libraries were prepared for sequencing on an Oxford Nanopore GridION (Oxford Nanopore Technologies (ONT), Oxford, UK) using the Rapid Barcoding Sequencing (SQK-RBK004) (ONT) kit where 333 ng template DNA was available per sample, using the manufacturer’s protocol. For low input samples (maximum 10 ng template DNA per sample) the Rapid PCR Barcoding kit (SQK-RPB004) (ONT) with modifications to the manufacturer’s protocol as reported by Charalampous et al. (20) and Street et al. (28) was used. Post-PCR, DNA samples were purified with AMPure XP SPRI beads (Beckman Coulter, High Wycombe, UK) and eluted in 10 mM Tris-HCl pH 8.0 with 50 mM NaCl. DNA was quantified with a Qubit 2.0 fluorometer. Between 95 and 99.6 fmol of combined library were loaded per flowcell. Samples were sequenced on ONT FLO-MIN106D (ONT v.R9.4.1) flow cells in batches of 6 samples per flow cell for clinical samples and 4 samples per flow cell for the clinical H. influenzae isolates.

### Pathogen specific PCR analysis.

Quantitative real-time PCR (qPCR) was performed to quantify relative abundance of H. influenzae, S. pneumoniae, S. aureus, M. catarrhalis and P. aeruginosa DNA within the total DNA extracts. Human Pathogen Detection Genesig kits and PrecisionPLUS 2x qPCR Mastermix by Primerdesign (Eastleigh, UK) were used as per manufacturer’s instructions. Primers and probes were used for the following bacterial targets: outer membrane protein P6 (H. influenzae), alpha fucosidase (S. pneumoniae), FEMB (S. aureus), major outer membrane protein/copB (M. catarrhalis) and toxin A synthesis regulating gene/RegA (P. aeruginosa). Reactions were performed in 20 μL with 5 μL template DNA (10 ng). Positive control templates for each bacterial species of interest (containing 2 × 10^5^ copies/μL) were used to prepare a standard curve dilution series ranging from 2 × 10^5^ to 20 copies/μL. Negative controls using nuclease free water (ThermoFisher, Waltham, MA, USA) in place of DNA template were used. All assays were performed in duplicate and mean values utilized in analyses. The qPCR conditions were: enzyme activation 2 min at 95°C followed by amplification for 50 cycles at 95°C for 10 s and 60°C for 60 s. qPCR assays were performed on a Bio-RadCFX96 instrument (Bio-Rad, Watford, UK).

### Bioinformatic and statistical analysis.

Statistical analysis was performed using GraphPad Prism (Version 9) and SPSS (Version 27.0). Parametric and nonparametric data are displayed as mean (SEM) and median (range), with comparisons between groups made using unpaired t tests or Mann‐Whitney tests, respectively.

Illumina; Raw sequencing reads were adapter trimmed using BBDuk (https://sourceforge.net/projects/bbmap/) and the adapter sequence file provided within the BBMap package; the following parameters were used: minlength, 36; k,19; ktrim, r; hdist, 1; mink, 12. Taxonomic classification of trimmed reads was performed using Centrifuge (35) and a database constructed from complete genomes of bacteria, viruses, and fungi uploaded to NCBI refseq as of 2019-08-24. The human genome (GRCh38) was included for host DNA detection. Haemophilus influenzae classified reads were mapped to reference genome NC_000907.1 using BWA MEM (version 0.7.12-r1039) (36) and SAMtools (version 1.9) (37).

Oxford Nanopore; Sequences were basecalled using Guppy (ONT, version 3.3.0+ef22818) and analyzed using the CRuMPIT pipeline (https://gitlab.com/ModernisingMedicalMicrobiology/CRuMPIT) (38) with the same Centrifuge database used for Illumina sequence analysis. This pipeline used Minimap2 (version 2.17-r954-dirty) (39) to align Haemophilus influenzae classified reads to reference genome NC_000907.1. A complete description of the bioinformatics used and secondary analysis with custom scripts to summarize data and generate plots is described in this GitLab repository (https://gitlab.com/ModernisingMedicalMicrobiology/asthmas-methods-paper-analysis). For comparison of H. influenzae genome between clinical samples and cultured strains consensus, fasta sequences were generated using Minimap2 and CLAIR2 using the genericbugONTworflow (https://gitlab.com/ModernisingMedicalMicrobiology/genericbugontworkflow) previously optimized and described (40). A SNP analysis was performed, and a distance matrix was generated using snp-dists (41).

### Data availability.

Raw FASTQ data is available on the European Nucleotide Archive with the project accession PRJEB47876.
