# Supplementary material for: Identifying Bacterial Airways Infection in Stable Severe Asthma Using Oxford Nanopore Sequencing Technologies
Source: Microbiol Spectr. 2022 Mar 24;10(2):e02279-21. doi: 10.1128/spectrum.02279-21 (PMC9045196; doi:10.1128/spectrum.02279-21)
Supplement: SUPPLEMENTAL FILE 2 — Supplemental material. Download SPECTRUM02279-21_Supp_2_seq15.pdf, PDF file, 1.4 MB [file spectrum02279-21_supp_2_seq15.pdf]

Identifying Bacterial Airways Infection in Stable Severe Asthma Using Oxford Nanopore Sequencing Technologies: Supplementary Material

Table S1: Clinical characteristics and airway inflammatory cell parameters of healthy control

Characteristic	Healthy control
**Sex**	Female
**Age (years)**	28
**BMI (kg/m^2)**	20.7
**Presence of atopy**	Nil
**Smoking history**	Ex-smoker
**Pack years**	10
**FEV1 (L)**	2.36
**FVC (L)**	2.78
**FEV1/FVC**	0.83
**FeNO (ppb)**	23
**Blood eosinophils ($x10^9$/L)**	0.16
**Sputum eosinophils (%)**	0
**Sputum neutrophils (%)**	53.2

Table S2: Sputum culture results; gold standard culture of induced sputum plug samples and speciation with MALDI-TOF

PID	Bacterial species identified on culture
1054	*Haemophilus influenzae, Streptococcus pneumoniae, Streptococcus parasanguinis, 2 unclassified species**
278	*Haemophilus influenzae, Streptococcus mitis, Streptococcus vestibularis*
133	*Haemophilus influenzae, Streptococcus pneumoniae, Streptococcus parasanguinis*
297	*Haemophilus influenzae, Streptococcus pneumoniae, Streptococcus vestibularis, 2 unclassified species**
288	*Haemophilus influenzae, Rothia mucilaginosa*
29	*Haemophilus influenzae, Streptococcus pneumoniae, Streptococcus salivarius, Rothia mucilaginosa*
214	*Haemophilus influenzae (clinical laboratory result)*
1049	*Streptococcus pneumoniae, Streptococcus agalactiae, Neisseria macacae, Streptococcus vestibularis, Streptococcus salivarius, Streptococcus oralis*
235	*Mixed upper respiratory tract flora (clinical laboratory result)*
306	*Streptococcus salivarius, Streptococcus parasanguinis, Rothia dentocarinosa, 2 unclassified species**
187	*Mixed upper respiratory tract flora (clinical laboratory result)*
1052	*Rothia mucilaginosa, Streptococcus parasangunis*
294	*Streptococcus vestibularis, Rothia mucilaginosa, Neisseria subflava, Streptococcus salivarius, Streptococcus parasanguinis, Neisseria flavescens*
1055	*Rothia mucilaginosa, Neisseria perflava, Streptococcus vestibularis, Streptococcus parasanguinis, Streptococcus salivarius*
1053	*Streptococcus mitis, Streptococcus sangunis, Streptococcus infantis*
1041	*Streptococcus pneumoniae, Streptococcus vestibularis, Rothia mucilaginosa, Streptococcus oralis*
5	*Rothia mucilaginosa, Streptococcus parasangunis, Streptococcus salivarius, Neisseria flavescens*
275	*Streptococcus pneumoniae, Streptococcus mitis, Streptococcus anginosus, 4 unclassified species**
315	*Streptococcus mitis, Streptococcus vestibularis, Neisseria flavescens, 3 unclassified species**
1060	*Streptococcus salivarius, Streptococcus parasanguinis, Streptococcus mitis*
279	*Streptococcus mitis, Streptococcus parasanguinis, Rothia dentocarinosa, Streptococcus vestibularis, Streptococcus salivarius, Streptococcus anginosus*
295	*Streptococcus salivarius, Rothia mucilaginosa, Streptococcus mitis, Streptococcus vestibularis*
1058	*Neisseria subflava, Neisseria flavescens, Streptococcus salivarius, Rothia dentocarinosa*

**Unclassified result on MALDI-TOF (including following formic acid treatment)*

Figure S1: Comparison of methods for depletion of human DNA with differential centrifugation and NEBNext microbiome DNA enrichment kit (n=2); (a) Percentage of total unfiltered reads by parameter detected using each DNA extraction method (mean, SD), (b) Total bacterial load measured by bacterial 16S RT-qPCR (mean, SD)

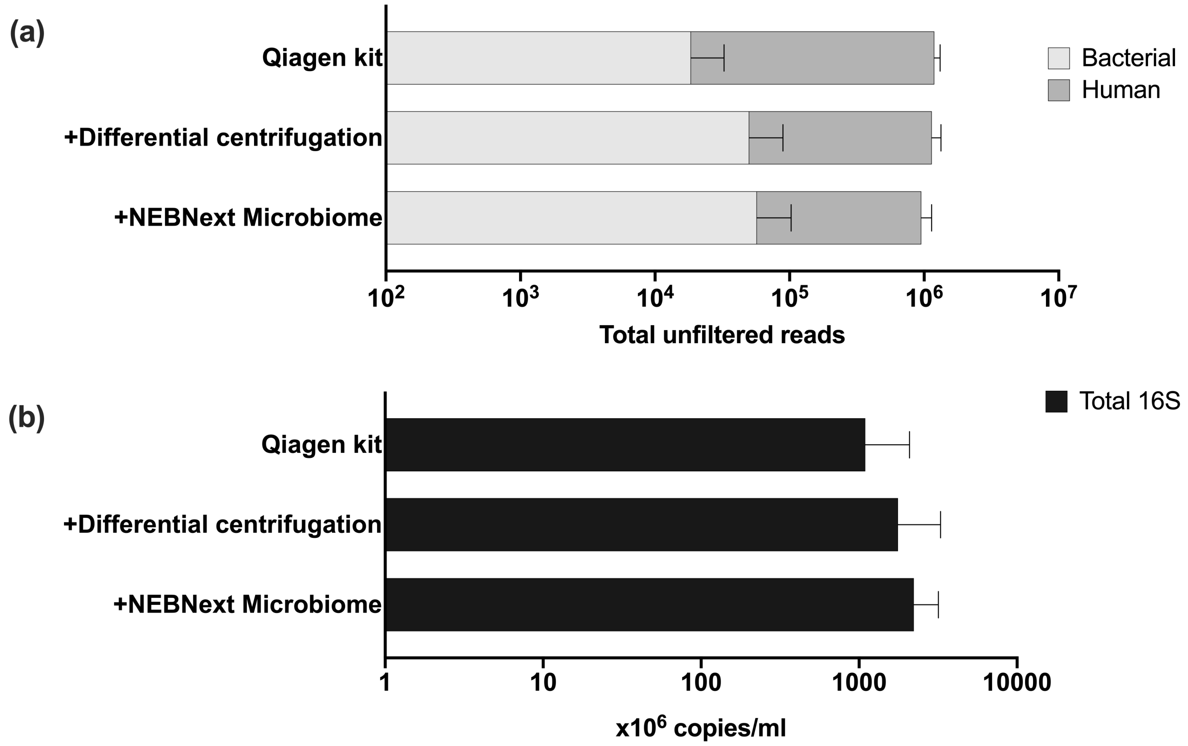

Figure S2: Total number of bacterial and human reads with (a) ONT and (b) Illumina MiSeq upon mapping all metagenomic sequencing reads. Total human reads are a sum of human reads removed following mapping to the human genome and residual reads subsequently detected by Centrifuge. This is plotted with the number human reads removed after mapping to the human genome in (c) and (d).

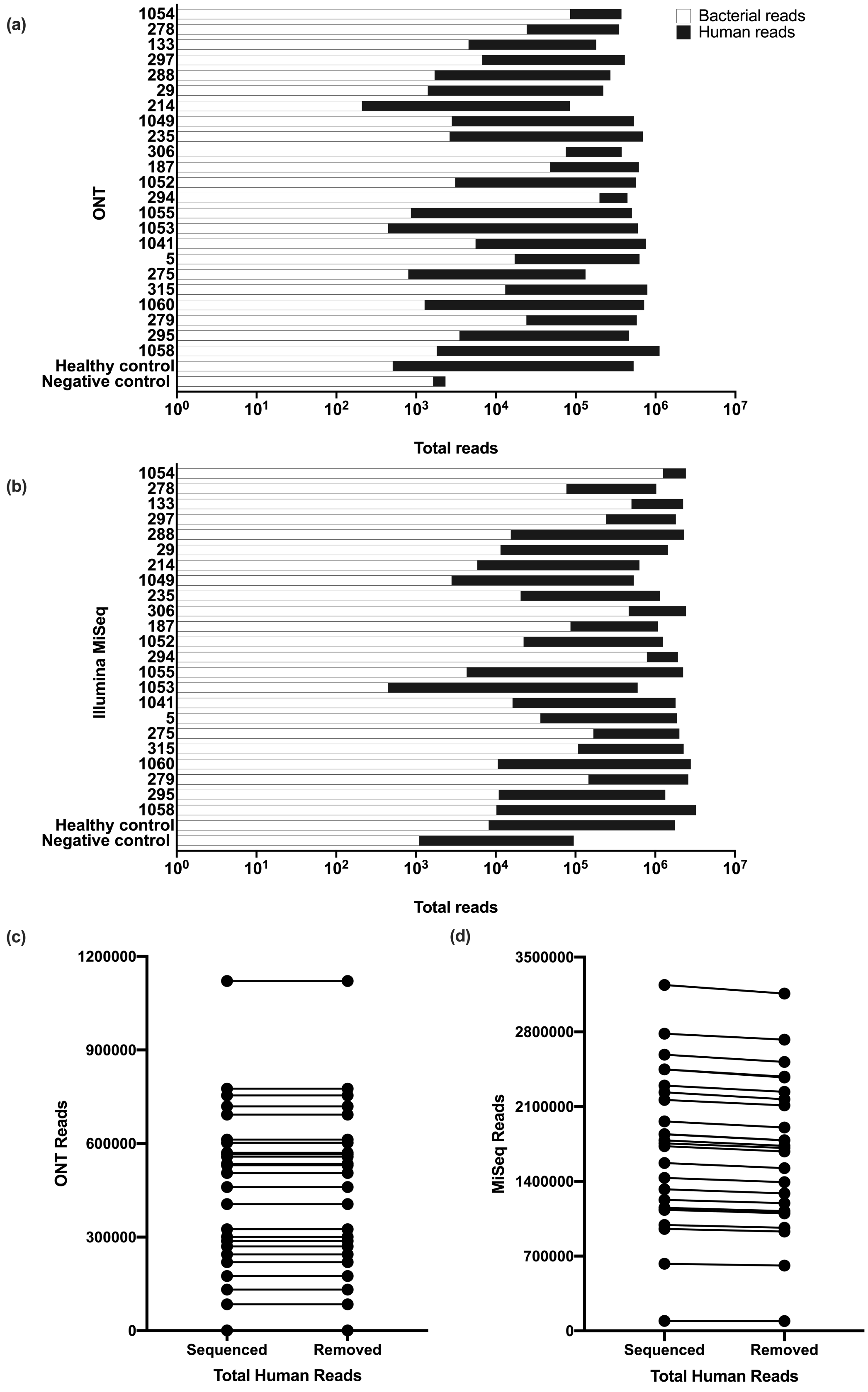

Table S3: Mapping coverage statistics of consensus fasta sequences; 4 clinical samples positive for H. influenzae (PID: 278, 133, 29, 288) and each corresponding bacterial isolate (*H.inf*_278, *H.inf*_133, *H.inf*_29, *H.inf*_288) are shown

Sample	As	Cs	Gs	Ns	Ts	Bases covered	% Covered	Length (bp)
PID 278	521515	317255	314865	157459	519044	1672679	91.4	1830138
H. inf_278	521365	321867	318231	149113	519562	1681025	91.9	1830138
PID 133	308467	184702	184786	846122	306061	984016	53.8	1830138
H. inf_133	521055	321265	318636	149903	519279	1680235	91.8	1830138
PID29	741	532	471	1827537	857	2601	0.1	1830138
H. inf_29	528329	325443	322892	126355	527119	1703783	93.1	1830138
PID 288	7581	4800	4415	1805539	7803	24599	1.3	1830138
H. inf_288	524167	323136	319242	141942	521651	1688196	92.2	1830138
